# Attention and Recall of Point-of-sale Tobacco Marketing: A Mobile Eye-Tracking Pilot Study

**DOI:** 10.3934/publichealth.2016.1.13

**Published:** 2016-01-12

**Authors:** Maansi Bansal-Travers, Sarah E. Adkison, Richard J. O'Connor, James F. Thrasher

**Affiliations:** 1 Department of Health Behavior, Roswell Park Cancer Institute, Buffalo, NY, USA; 2 Department of Health Promotion, Education & Behavior, Arnold School of Public Health, University of South Carolina, Columbia, SC, USA

**Keywords:** mobile-eye tracking, tobacco advertising, smoking, point-of sale

## Abstract

**Introduction:**

As tobacco advertising restrictions have increased, the retail ‘power wall’ behind the counter is increasingly invaluable for marketing tobacco products.

**Objective:**

The primary objectives of this pilot study were 3-fold: (1) evaluate the attention paid/fixations on the area behind the cash register where tobacco advertising is concentrated and tobacco products are displayed in a real-world setting, (2) evaluate the duration (dwell-time) of these fixations, and (3) evaluate the recall of advertising displayed on the tobacco power wall.

**Methods:**

Data from 13 Smokers (S) and 12 Susceptible or non-daily Smokers (SS) aged 180–30 from a mobile eye-tracking study. Mobile-eye tracking technology records the orientation (fixation) and duration (dwell-time) of visual attention. Participants were randomized to one of three purchase tasks at a convenience store: Candy bar Only (CO; N = 10), Candy bar + Specified cigarette Brand (CSB; N = 6), and Candy bar + cigarette Brand of their Choice (CBC; N = 9). A post-session survey evaluated recall of tobacco marketing. Key outcomes were fixations and dwell-time on the cigarette displays at the point-of-sale.

**Results:**

Participants spent a median time of 44 seconds during the standardized time evaluated and nearly three-quarters (72%) fixated on the power wall during their purchase, regardless of smoking status (S: 77%, SS: 67%) or purchase task (CO: 44%, CSB: 71%, CBC: 100%). In the post session survey, nearly all participants (96%) indicated they noticed a cigarette brand and 64% were able to describe a specific part of the tobacco wall or recall a promotional offer.

**Conclusions:**

Consumers are exposed to point-of-sale tobacco marketing, regardless of smoking status. FDA should consider regulations that limit exposure to point-of-sale tobacco marketing among consumers.

## 1. Introduction

The tobacco industry spent over 8.5 billion dollars a year on marketing and advertising for their products in the United States in 2010, 84% of which was spent in the retail environment. Of that marketing budget, 94% was spent on strategies specifically at the point-of-sale[1]. The industry has historically made concerted efforts to entice young people to use tobacco products with promotions and discounts. These marketing efforts encourage young people to take up smoking[2] and are prominently displayed in retail environments at the point-of-sale[3]. The concentrated tobacco marketing efforts represent a powerful advertising campaign targeted towards this susceptible population.

The role of point-of-sale marketing and advertising in increasing appeal and intention to use tobacco among youth is well documented[3]–[13], as is its influence on unintended purchases and undermining quit attempts among current and former users.[5],[14]–[18]. These findings have prompted lawmakers to ban tobacco point-of-sale marketing in some jurisdictions in Australia, Canada, and Iceland, among others which has resulted in a reduction of spontaneous purchases, as well as considerable declines in awareness of tobacco promotions among smokers[14]. Despite this, point-of-sale tobacco marketing remains a prominent feature, particularly in convenience stores, drug stores, and gas stations in the United States.

While considerable research documents the effect of exposure to tobacco marketing, the measures are typically self-reported frequency of visiting stores and awareness of marketing, or participants' proximity to stores with point-of-sale advertising[5]. Presence or absence of point-of-sale advertising has been manipulated in two experimental studies that exposed youth to photos of the retail environment[7],[12] and in two experiments where participants were given a shopping task within a virtual retail environment[13],[19]. However, no study to date evaluates quantitative measures of the duration of attention paid to advertising and promotional materials at the point-of-sale in a real-world (or virtual) retail environment.

**Figure 1.**
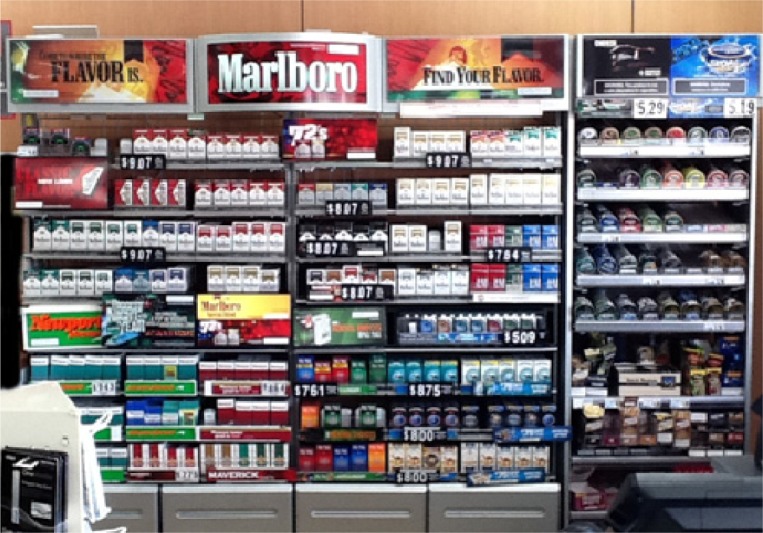
Example of the tobacco power wall at the point-of-sale in the U.S. at time of study.

The current research aims to expand the research on the impact and value of point-of-sale marketing and advertising for tobacco products. Specifically, this pilot study assessed how young adults interact with point-of-sale tobacco advertising in a real-world retail environment with three primary objectives: (1) examine the attention paid/number of fixations on the area behind the cash register where tobacco advertising is concentrated and tobacco products are displayed (i.e. the tobacco ‘power wall’; see Figure 1), (2) evaluate the duration (dwell-time) of these fixations, and (3) evaluate the recall of advertising displayed on the tobacco power wall.

## 2. Materials and Methods

### 2.1. Procedure

Participants were recruited in the Buffalo, NY, area via flyers and word of mouth for a pilot study. Participants were eligible if they were between 18 and 30 years old, able to read and write in English, and able to meet the researchers at one of four selected convenience store locations. Locations were selected to reflect similar environments such that point-of-sale advertising comprised comparable space and dimensions across locations. Participants included both smokers (S; i.e. ‘every day’) and susceptible or non-daily smokers (SS; i.e. ‘some days over the past 30 days’). Susceptibility was defined as responding ‘definitely yes’, ‘probably yes’, or ‘probably not’ to the following four questions: “Do you think you will smoke a cigarette soon?”; “Do you think you will smoke a cigarette in the next year?”; “Do you think that in the future you might experiment with cigarettes?”; and “If one of your best friends were to offer you a cigarette would you smoke it?”. Participants were excluded if they wore hard contact lenses, hard-lined bifocal or trifocal glasses, or colored contact lenses because they are extremely difficult to reliably eye-track due to interruption in the reflection pathway[20]. This study was approved by the Roswell Park Cancer Institute Institutional Review Board.

At the start of the session, at a separate retail location near the convenience store, researchers briefly explained the study procedures and participants provided oral consent. Written consent was not obtained because there was no more than minimal risk to participants for partaking in this research. Participants then completed a ten-minute baseline survey on their smoking history and habits, current craving, beliefs about smoking and health, and intention to quit (among current users). Following the baseline questionnaire, participants were fitted and calibrated to the mobile eye-tracking system (Applied Science Laboratories Mobile Eye-XG), consisting of a lightweight pair of spectacles (2.75 ounces) that hold a tiny digital camera and infrared light source. The light reflects off the cornea and the camera records the reflected light, enabling the tracking of the pupil. Specialized equipment and software links pupil and head position with regions of interest in a “scene” (what the participant is looking at) and records it. After being fitted to the system, participants were asked to look around in their environment to ensure proper calibration and acclimation to the equipment. Once comfortable with the equipment, participants were given cash and asked to enter the convenience store and purchase specific items based on their pre-randomized purchase task group. Participants were randomized to one of three purchase tasks: Candy bar Only (CO), Candy bar and a Specified cigarette Brand (CSB), and Candy bar and a cigarette Brand of Choice (CBC). These three conditions were selected to evaluate differences between how consumers may interact with and vary in time spent looking at the power wall. After completing the purchase task, participants exited the store, met the researchers, and returned to the site of calibration to complete a post-session survey evaluating recall of store ads and displays, current craving, and perceptions of tobacco marketing. Participants were not allowed to keep the items purchased; those who completed the 25–30 minute session were mailed a $30 check.

A total of 30 participants were recruited for the study and real-time video recordings were made of each participant's mobile eye-tracking data from entrance to exit of the convenience store. Five participants' data were eliminated from the analysis; one would not calibrate the system properly (astigmatism) and four had video data that could not be mapped to the retail environment (glare in the recorded video or distance and angle from the point-of-sale prevented proper mapping). Therefore, data from 25 participants were included in the analysis (CO, N = 10; CSB, N = 6; CBC, N = 9). Figure 1 presents an example of the retail environment from this study. Each location devoted the same amount of retail space to tobacco products, though exact placement and number of advertisements and promotional materials at the point-of-sale varied slightly by location. However, pictures were taken at each of the four locations at the beginning and end of the study period and the content of the displays remained fairly constant throughout.

### 2.2. Measures

#### 2.2.1. Fixations

Fixation data was based on “dwell-time fixation detection,” such that a fixation begins when a participant orients their point of gaze in a particular area for the threshold of cognition to occur. The threshold of cognition is the point at which the eye orients and remains within 1-degree visual angle for at least 0.1 second or 100 milliseconds[21]. The 1-degree minimum change was selected because minute eye movements such as tremors, drifts, and micro-saccades are typically less than 1 degree[22].

#### 2.2.2. Dwell time

Dwell time is the time that an individual spends looking in a particular area of interest fixating in that area without leaving. This allows us to better evaluate the overall “interaction with the areas of interest,” not with the specific fixation events within them. Percent of time participants spent focusing on the tobacco display area at the point-of-sale is presented.

### 2.3. Statistical Analysis

The retail environment was first “mapped” at each location, which allows for the participants' real-time video data to be used to calculate fixations and dwell times in specifically defined Areas of Interest (AOIs). The environments were mapped using Applied Science Laboratories Results Plus analysis software[22]. Mapping confidence above 75% is considered to be a “good” map and allows for adequate detection of fixations and dwell time. Each location mapped with a high level of confidence (range: 92.4%–99.3%); Table 1 displays the percent confidence at each of the four locations, number of total participants tracked at each location, and the average time spent in the store.

**Table 1.** Location Information.

Location	Number of Participants	% Confidence Mapping	Median Time Spent from Item to check out in sec (Min-Max)
**1**	16	97.5	49.91 (4.81–321.08)
**2**	3	92.4	13.69(5.91–51.59)
**3**	3	99.3	22.71 (18.97–43.78)
**4**	3	99.2	56.03 (5.71–60.07)
	N = 25	AVG = 97.1%	Median time in store: 43.78

Once the map was created, AOIs were defined within the retail environment: (1) Tobacco advertising, which included basic ads for tobacco products as well as price promotions or discounts, (2) Cigarette displays, which included displays of the cigarette packs on the power wall, and (3) Smokeless tobacco products, which included all smokeless tobacco products including spit and no-spit (snus) products. E-cigarettes were not evaluated because they were not prominently advertised or displayed at the point-of-sale in any of the locations at the time of this study (see Ganz, et al. for a discussion of electronic cigarettes at the point-of-sale[23]). Definition of these AOIs allowed us to evaluate the percent of time that participants dwelled within each AOI. Although specific ads and displays were not the same across the four stores, the AOIs evaluated in this pilot study were mapped to each store individually, and those areas remained constant through the study period. Total time period of interest from each participant's data was standardized to include the time where exposure to the power wall was possible and therefore was standardized to the period of time from when the participant selected the candy bar until the candy bar was rung up at the checkout.

The results are presented in four sections. First, sample characteristics and a brief overview of the purchase task experience; second, aggregate data for the overall fixation data for total time participants spent in the retail environment (because participants spent a widely varied amount of time in the retail environment during their purchase experience); third, one-way ANOVAs comparing point-of-sale exposure time by condition at the individual level for the percent of fixations and dwell time in the AOIs. Bonferroni corrections were used to identify differences in percent of exposure time by purchase-task condition. T-tests were used to identify differences in exposure time based on smoking status. Finally, participants' recall of the tobacco power wall and advertising is presented.

## 3. Results

### 3.1. Sample Characteristics

Overall, 13 participants were smokers (52%) (median age 23.6 years), and 12 (48%) were non-daily or susceptible smokers (median age 25.6 years; Table 2). Participants did not differ significantly by age, sex, or smoking status, and all reported having smoked a cigarette at some point in their lives. Thirteen percent reported visiting a convenience store, gas station, or liquor store where tobacco advertising is typically present nearly every day, 48% reported going 2–3 times a week, and 40% reported going at least once a week.

Participants spent a median time of 44 seconds during the standardized time period (i.e. time from selection of candy bar to cash out) while in the retail environment. Nearly three-quarters (72%) of fixated on the power wall at some point during this period (S: 77%, SS: 67%; CO: 44%, CSB: 71%, CBC: 100%). Those in the CO condition were less likely than the CBC condition to fixate on the power wall (χ^2^ (2, *N* = 19) = 7.892, *p* = 0.011).

**Table 2.** Sample Characteristics.

		All	Smoker (S, N = 13)	Susceptible Smoker (SS, N = 12)
**Sex**	Male	56%	46%	66%
	Female	44%	54%	33%
**Age**	18–21	32%	39%	25%
	22–25	36%	39%	33%
	26–30	32%	23%	42%
**Hispanic**	Yes	4%	0%	8%
	No/DK	96%	100%	92%
**Race**	White	96%	100%	92%
	Other	4%	0%	8%
**Education**	≤ 12	24%	23%	25%
	> 12	76%	77%	75%
Tried to quit in the last year*	Yes	72%	54%	92%
	No	28%	46%	8%
**Visit**	Every day	13%	23%	0%
**Store**	2–3x week	48%	54%	40%
	1x week	39%	23%	60%

*Significant difference detected between groups, Chi-square statistic, *p* < 0.05

### 3.2. Aggregate fixation and dwell time

To determine the attention paid/fixations on the AOIs on the power wall, number and percent of overall fixations during the study session were totaled for all participants. The majority of fixations during this period were outside of the areas of interest, although 21% included the tobacco power wall at the checkout. Susceptible smokers (18.3%) and women (18.3%) tended to have more fixations on the cigarette packs than smokers (10.9%) and men (12.4%). Smokers (8.2%) and men (7.0%) tended to have more fixations on advertisements than susceptible smokers (2.8%) and women (2.9%). Fixations tended to be most common among participants randomized to the CBC condition (27.9%), followed by the CSB condition (21.9%) and finally the CO condition (7.8%). Table 3 presents the aggregate number and percent of overall fixations on cigarette packs, ads, and smokeless tobacco products by smoking status, sex, and purchase task.

**Table 3.** Total number of fixations in AOIs overall, by smoking status, sex, and purchase task.

		Outside AOI	Cigarettes	Ads	ST	Total Wall (inside AOI)
		N(%)	N(%)	N(%)	N(%)	N(%)
**All**	All	2098 (79.2)	374 (14.1)	155 (5.9)	21 (0.8)	550 (20.8)
						
**Smoking Status**	Smoker	1199 (80.0)	164 (10.9)	123 (8.2)	13 (0.9)	200 (20.0)
	Susceptible Smoker	899 (78.2)	210 (18.3)	32 (2.8)	8 (0.7)	250 (21.8)
						
**Sex**	Male	1504 (79.6)	235 (12.4)	133 (7.0)	17 (0.9)	385 (20.3)
	Female	594 (78.3)	139 (18.3)	22 (2.9)	4 (0.5)	164 (21.7)
						
**Purchase Task**	CO	708 (92.2)	29 (3.8)	23 (3.0)	8 (1.0)	60(7.8)
	CSB	450 (78.1)	106 (18.4)	16 (2.8)	4 (0.7)	126 (21.9)
	CBC	940 (72.1)	239 (18.3)	116 (8.9)	9 (0.7)	364 (27.9)

Note: AOI, Area of Interest, CO: Candy Only, CSB: Candy and Specific cigarette Brand, CBC: Candy and cigarette Brand of Choice, ST: smokeless tobacco; no significant differences were identified between locations for the number and percent of fixations on cigarettes, advertisement, and smokeless tobacco

In the aggregate, participants spent over one-quarter (27.6%) of their total dwell time within fixations on AOIs containing tobacco advertisements, cigarettes, and smokeless tobacco at the point-of-sale. This tended to be somewhat more common among smokers (29.4%) than susceptible smokers (24.9%) and among males (28.4%) than females (25.1%). Those randomized to the CBC condition (i.e. where the participant could select a cigarette brand of choice) tended to spend more dwell-time on tobacco-related items at the point-of-sale (38.2%), than those who purchased a specific brand (25.8%) or candy alone (8.2%). Table 4 outlines the percent of total dwell time spent in each AOI by smoking status, sex, and purchase task.

### 3.3. Individual level fixation and dwell time

At the individual level, t-tests showed no significant differences by smoking status or sex on the number or percent of fixations that included the power wall (Table 5). Analysis of variance showed no difference between conditions for the number of fixations on cigarettes, advertisements, and smokeless tobacco; however, the total percent of fixations on the AOIs at the point-of-sale varied significantly by purchase task. Bonferroni-corrected comparisons showed that participants in the CO condition had a lower percent of total fixations on the tobacco power wall (*F* (2, 22) = 4.937, *p* = 0.017), and on cigarettes in particular (*F* (2, 22) = 5.051, *p* =0.016), than those in the CBC condition during their time in the store.

T-tests showed that there were no differences by smoking status or sex on percent of dwell time on the tobacco power wall. Analysis of variance showed that there were no differences by condition for viewing ads and smokeless tobacco products, though those in the CBC and CSB condition spent a greater percent of time dwelling on the cigarette packs (*F* (2, 22) = 5.476, *p* = 0.012), and the power wall in general (*F* (2, 22) = 4.903, *p* = 0.017).

**Table 4.** Percent of dwell time in AOIs overall, by smoking status, sex, and purchase task.

		Outside AOI	Cigarettes	Ads	ST	Total Wall (Inside AOI)
**All**	All	72.4%	18.0%	8.8%	0.8%	27.6%
						
**Smoking Status**	Smoker	70.6%	15.4%	13.1%	0.9%	29.4%
	Susceptible Smoker	75.1%	21.8%	2.5%	0.6%	24.9%
						
**Sex**	Male	71.6%	16.7%	10.8%	0.9%	28.4%
	Female	74.9%	21.9%	2.7%	0.5%	25.1%
						
**Purchase Task**	CO	91.8%	3.7%	3.4%	1.1%	8.2%
	CSB	74.2%	22.6%	2.5%	0.7%	25.8%
	CBC	62.3%	23.7%	13.8%	0.7%	38.2%

Note: AOI: Area of Interest, CO: Candy Only, CSB: Candy and Specific cigarette Brand, CBC: Candy and cigarette Brand of Choice, ST: Smokeless Tobacco

**Table 5.** Individual level percent of dwell time in AOIs overall, by smoking status, sex, and purchase task.

		Outside AOI	Cigarettes	Ads	ST	Total Wall (Inside AOI)
**All**	All	76.5%	19.3%	3.2%	1.0%	23.5%
						
**Smoking Status**	Smoker	80.6%	14.0%	4.0%	1.4%	19.4%
	Susceptible	72.1%	25.1%	2.4%	1.0%	27.9%
	Smoker					
**Sex**	Male	77.8%	16.5%	4.1%	1.5%	22.2%
	Female	74.8%	22.9%	2.1%	0.0%	25.2%
						
**Purchase Task**	CO	93.7%	3.0%	2.5%	1.0%	6.3%
	CSB	67.3%	28.4%	3.8%	0.0%	32.7%
	CBC	63.5%	31.4%	3.7%	1.4%	37.5%

Note: CO: Candy Only, CSB: Candy and Specific cigarette Brand, CBC: Candy and cigarette Brand of Choice, ST: Smokeless Tobacco

### 3.4. Tobacco Display Recall

In the post-session survey, administered upon exit from the convenience store, 96% of participants indicated that they noticed a cigarette brand and 64% were able to describe a specific part of the tobacco wall or recall a promotional offer (S: 77%, SS: 50%; CO: 70%, CSB: 17%, CBC: 89%). For example, one respondent [correctly] reported that “Marlboro's $1 off a pack when you buy two packs” and another recalled that “There was an appealing display on the right side behind the cashier.” Participants in the CSB condition were less likely than the CBC condition to recall advertising that caught there attention at the point-of-sale (χ^2^ (1, *N* = 15) = 7.824, *p* = 0.005). No differences were identified in cravings following the purchase task among smokers. Additionally, 64% of participants indicated that, in the past 30 days, they were exposed to tobacco advertising in a convenience store, gas station, or supermarket either ‘most of the time’ or ‘always’.

## 4. Discussion

In the current pilot study, mobile eye-tracking technology was used to assess consumers' real-time duration of exposure time to marketing elements within a retail environment, allowing us to evaluate how consumers interact with advertising and promotional materials at the point-of-sale. The mobile eye-tracking technology provides the first quantitative measure of exposure time that complements the qualitative data collected through recall of advertising at the end of the session. Our results show the overwhelming majority of participants were exposed to the tobacco power wall, regardless of smoking status, and that quantitatively-measured exposure time to the wall was high, with 44% of participants who were randomized to the condition where they were instructed to only purchase a candy bar fixating on the power wall at some point.

While mobile eye-tracking identifies only where participants are fixating and dwelling in those areas, it is unable to tell us whether or not individuals are processing information in that region. However, when participants were asked to recall their shopping experience, over half of participants were able to identify a specific promotion or other aspect of the tobacco power wall at the point-of-sale. Seventy percent of those in the CO condition recalled an ad, suggesting that even consumers who do not intend to purchase tobacco products experience exposure to promotional materials and advertising while in the retail environment

In evaluating these results, some limitations should be considered. First, participants were a convenience sample of young adult smokers and susceptible/non-daily smokers, limiting the generalizability of the findings to the broader public. Furthermore, the sample size was too small and uneven across conditions (due to data mapping issues) to assess small to medium effect sizes and to eliminate selection bias through random allocation. While t-tests indicated there were no differences in attention paid to tobacco advertising at the point-of-sale by smoking status or sex, it may be that differences will be revealed with a larger sample. This pilot study demonstrated that this work is feasible in the real-world environment, while also providing preliminary data for a larger study that might address some of the limitations presented.

Participants also met us at one of four different locations of their choosing for convenience. While locations provided a similar environment and video time was standardized to only the time frame from candy bar selection to check out, individual stores may not present consumers with similar shopping experiences (e.g. slight differences in cigarette and promotional materials at checkout). Additionally, because participants were instructed to make a purchase during operating retail hours it was not possible to control the overall environment (e.g. time spent completing the task). While this represents an actual, more naturalized, shopping experience rather than a controlled or virtual setting, it likely introduced some error. For example, shopping while wearing the eye-tracking apparatus may not be the same as shopping without wearing the mobile eye-tracking technology—both in participants' own behavior and how others (e.g. other shoppers, store employees) react to them. However, review of the video after each participant showed that, although others did occasionally ask participants about the apparatus, the participants reacted fairly naturally and did not seem to deviate significantly from the task at hand. Therefore, wearing the apparatus likely did not significantly impact the experience or the results.

Finally, at times, the software package was unable to properly map participants' gaze to the areas of interest at the point-of sale. This resulted in a portion of data to go unrecorded. For example, occasionally participants focused on tobacco advertising at the point-of-sale, but because the angle to the power wall was too sharp or the respondent was too far away, the times spent in those locations were not considered for the analysis. This likely resulted in conservative estimates for the overall exposure time to the defined areas of interest.

## 5. Conclusions

This study fills an important gap in the literature regarding exposure time to tobacco advertising at the point-of-sale. In a systematic review of the research on advertising at the point-of-sale, Paynter and Edwards (2009) note that much of the previous research has been unable to accurately quantify the effect of tobacco marketing. Recent work published by Kim et al. summarized a virtual reality experiment conducted with varying manipulations of the point-of-sale display and the retail advertising[13],[19]. Although their work found that enclosing the point-of-sale display lowered the likelihood that youth will try to purchase tobacco, these findings are in the context of the virtual store. Furthermore, it does not assess amount of exposure time, which mobile eye-tracking allows. This study extends the generalizability of this type of experiment by applying the purchase task method to an actual store environment, where the exposure time and experience is more realistic. Our results show that, regardless of purchase condition or smoking status, consumers experience exposure time to tobacco advertising and promotional materials at the point-of-sale. Because exposure has been shown to influence youth uptake and impulse purchases among smokers and those trying to quit[5], the findings from the current research highlight the critical importance of implementing regulations that restrict tobacco advertising, marketing, and product displays at the point-of-sale.
